# Comprehensive Analysis of the Immune Cell Infiltration Landscape and Immune-Related Methylation in Retinoblastoma

**DOI:** 10.3389/fgene.2022.864473

**Published:** 2022-05-18

**Authors:** Peiyao Mao, Yinchen Shen, Xun Xu, Jiawei Zhong

**Affiliations:** ^1^ Shanghai Key Laboratory of Ocular Fundus Diseases, Department of Ophthalmology, Shanghai General Hospital, School of Medicine, Shanghai Jiao Tong University, National Clinical Research Center for Eye Diseases, Shanghai Engineering Center for Visual Science and Photomedicine, Shanghai Engineering Center for Precise Diagnosis and Treatment of Eye Diseases, Shanghai, China; ^2^ State Key Laboratory of Ophthalmology, Zhongshan Ophthalmic Center, Sun Yat-Sen University, Guangzhou, China

**Keywords:** retinoblastoma, immune cell infiltration, DNA methylation, bioinformatics analysis, immunotherapy

## Abstract

Retinoblastoma is a common pediatric intraocular cancer, originating from cone precursors. The development of immunotherapies can help eradicate the tumor without vision loss, which would largely improve the quality of life of patients with retinoblastoma. Investigation of the tumor immune microenvironment provides knowledge for developing novel immunotherapies in cancer. However, the immune cell infiltrative landscape of retinoblastoma is unknown. Here, we compared the relative expression of immune gene signatures among 59 patients with retinoblastoma. The patients were divided into two subgroups according to the 28 types of immune cell infiltration (ICI) scores. We found that a subgroup with high ICI scores had increased expression levels of late cone markers, while the other subgroup exhibited larger tumor size and metastasis propensity. Furthermore, hypermethylated genes in the high-ICI subgroup were associated with immune regulation in the tumor microenvironment, suggesting that DNA methylation may play a vital regulatory role in retinoblastoma immunity. Our study provides a comprehensive framework for the systemic analysis of the influences of epigenetic events on the tumor immune microenvironment. We anticipate that our assay can not only provide insights into tumor immune regulation but also open up the perspectives for the identification of novel immunotherapy targets for retinoblastoma.

## Introduction

Retinoblastoma is one of the most common primary ocular malignancies in children with an incidence of 1:16000–1:18000 (Dimaras et al., 2015). It is usually initiated by biallelic retinoblastoma gene (*RB1*) mutation. Despite the significant improvement in treatments including cryotherapy, radiotherapy, ophthalmic artery chemosurgery, and intravitreous chemotherapy, some retinoblastoma patients eventually develop metastases due to invasion of the central nervous system through the optic nerve and dissemination through the sclera to the orbit (Gündüz et al., 2006; Abramson et al., 2015; Lu et al., 2019). Over the past several decades, cancer immunotherapy, including immune checkpoint blockade, vaccination, and adoptive T-cell therapy, has brought significant improvement for patients in terms of survival and quality of life (Esfahani et al., 2020). However, compared with other cancer types, few immunotherapies have been applied to patients with retinoblastoma (Schefler and Kim, 2021). Therefore, a systematic investigation of the tumor immune microenvironment is essential for the development of effective anti-tumor immunotherapies.

With the help of novel technologies such as single-cell RNA sequencing (scRNA-seq), the critical role of the tumor microenvironment (TME) in tumor genesis, invasion, metastasis, and relapse has been revealed (Schelker et al., 2017; Steele et al., 2020; Lim et al., 2021; Mani et al., 2022). The TME in retinoblastoma contains numerous immune cells, including dendritic cells, monocytes, macrophages, and T-lymphocyte cells (Sarver et al., 2021). For example, a previous study has shown that reduced retinoblastoma cell proliferation was associated with increased immune cell infiltration (Sarver et al., 2021). Moreover, bioinformatics algorithms are developed for an immune infiltration estimation of a series of cancer tissues based on their transcriptional data (Hänzelmann et al., 2013; Yoshihara et al., 2013; Newman et al., 2015). These methods have significantly promoted our understanding of the TME and have been applied to hepatocellular carcinoma (Liu S. et al., 2021), clear cell renal cell carcinoma (Zhang et al., 2021), pancreatic adenocarcinoma (Liu et al., 2020), and so on. However, the application of retinoblastoma has not been fully elucidated.

DNA methylation has proved its role as the crucial epigenetic regulator in cancer progression by regulating genome sequence stability and gene expression (Feinberg et al., 2016). It is commonly known that the inactivation of certain tumor-suppressor genes occurs as a consequence of hypermethylation within the promoter regions (Kulis and Esteller, 2010). Moreover, abnormal methylation events were observed in retinoblastoma (Stirzaker et al., 1997; Berdasco et al., 2017). However, the relationship between DNA methylation and the immune microenvironment of retinoblastoma has not been broadly interrogated.

In this study, based on the immune profile of 28 types of immune cells, we identified two immunological subgroups of retinoblastoma. These two subgroups of retinoblastoma patients have distinct clinical characteristics and gene expression profiles. Next, we systematically examined the distinct DNA methylation patterns between these two subgroups. Moreover, we screened 6 differentially methylated and expressed genes as hub genes, which may provide new insights into the molecular pathogenesis and the clinical immunotherapy of retinoblastoma.

## Materials and Methods

### Data Collection From GEO Databases

Gene expression arrays from 59 samples diagnosed with retinoblastoma were obtained from GEO databases with accession code GSE58780. The DNA methylation array from retinoblastoma patients was obtained from GEO databases with accession code GSE58783. Clinical data of all samples were downloaded from https://static-content.springer.com/esm/art%3A10.1038%2Fs41467-021-25792-0/MediaObjects/41467_2021_25792_MOESM4_ESM.xlsx. scRNA-seq data from retinoblastoma patients were obtained from GEO databases with accession code GSE174200.

### Immune Cell Infiltration Analysis

We performed a single-sample Gene Set Enrichment Analysis (ssGSEA) by using the *GSVA* (version 1.34.0) (Hänzelmann et al., 2013) R package based on the default parameters to calculate the immune infiltration level of 28 immune cell types (Charoentong et al., 2017). Among these immune cells, the activated CD4^+^ T cell, activated CD8^+^ T cell, central memory CD4^+^ T cell, central memory CD8^+^ T cell, effector memory CD4^+^ T cell, effector memory CD8^+^ T cell, type 1 T helper cell, type 17 T helper cell, activated dendritic cell, CD56^bright^ natural killer cell, natural killer cell, and natural killer T cell are considered to have anti-tumor capacities. Regulatory T cell, type 2 T helper cell, CD56^dim^ natural killer cell, immature dendritic cell, macrophage, MDSC, neutrophil, and plasmacytoid dendritic cell are considered to have pro-tumor capacities. We also used the Estimation of STromal and Immune cells in MAlignant Tumors using Expression data (ESTIMATE) algorithm of the *estimate* (version 1.0.13) (Yoshihara et al., 2013) R package to calculate the stromal and immune scores and tumor purity of each sample.

### Clustering Analysis Based on Immune Cell Infiltration Analysis

By using the *stats* (version 3.6.0) R package, we performed an unsupervised hierarchical clustering (based on Euclidean distance and Ward’s linkage) to cluster retinoblastoma samples based on a sample-signal matrix including 28 types of immune cells of 59 retinoblastoma samples. Fifty-nine samples were divided into high and low infiltration subgroups. The visualization of K-means clustering result was performed by the *pheatmap* (version 1.0.12) R package, and comparison between the two subgroups in terms of their signal enrichment score of 28 immune cell types was computed using a two-sided t-test and visualized by the *ggpubr* (version 0.3.0) R package.

### Principal Component Analysis

We performed a principal component analysis (PCA) on the sample-signal matrix using *FactoMineR* (version2.4) R package with default parameters. The result from the PCA was visualized by the *factoextra* (version 1.0.7) R package.

### Differential Expression Analysis

The sample-gene gene expression matrix was input into the *limma* (version 3.42.2) (Ritchie et al., 2015) R package for the identification of differentially expressed genes between the high-ICI subgroup and low-ICI subgroup. We determined differentially expressed genes (DEGs) with the criteria of absolute fold change >1.5 and false discovery rate (FDR) adjusted *p* < 0.05. Clusters of DEGs were identified by an unsupervised hierarchical cluster analysis (based on Euclidean distance and Ward’s linkage).

### Enrichment Analysis

An enrichment analysis was performed using the *clusterProfiler* (version 3.14.3) (Yu et al., 2012) R package with the “*enricher*” and “*GSEA*” function, and the FDR adjusted to *p* < 0.05 was considered as statistically significant. All gene sets were obtained from the Molecular Signatures Database (MSigDB) using the *msigdbr* (version 7.2.1) R package.

### DNA Methylation Array Processing and Differential Methylation Analysis

After obtaining the microarray data from GSE58783, we used the “*champ.filter*” function of the *ChAMP* (version 2.16.2) (Tian et al., 2017) R package to remove probes which are located in sex chromosomes and near SNP to eliminate the influence of sex and SNP, respectively. We used the “*champ.DMP*” function of the *ChAMP* R package with the criteria of absolute Δβ > 0.2 and FDR adjusted *p* < 0.05 for the identification of differentially methylated probes (DMPs) between the high-ICI subgroup and low-ICI subgroup. We next excluded the genes which had both hypermethylated and hypomethylated probes. Genes with either a hypermethylated probe or hypomethylated probe were considered as hypermethylated genes or hypomethylated genes, respectively.

### Protein–Protein Interaction Network

We used *STRING* (version 11.5) (Szklarczyk et al., 2019) with default parameters to construct the protein–protein interaction (PPI) network. The generated PPI networks were visualized by *Cytoscape* software (version 3.9.0) (Shannon et al., 2003). In *Cytoscape*, we used *cytoHubba* (Chin et al., 2014) to screen hub genes by the Maximal Clique Centrality (MCC) method.

### Statistical Analysis

All analyses were performed by R software (version 3.6.0). An unpaired two-tailed *t*-test was used to compare two subgroups of continuously distributed variables. The correlations of the retinoblastoma subgroups and clinical characteristics were analyzed using the chi-square test. *p* ≥ 0.05 (n.s.), *p* < 0.05 (*), *p* < 0.01 (**), *p* < 0.001 (***), and *p* < 0.0001 (****).

### Code Availability

All custom computer codes used in this study are freely available at https://github.com/jiawei-zhong/Mao_et_al_RB/

## Results

### Identification of Immune-Related Gene Subtypes in Retinoblastoma Based on Immune Cell Infiltration

**FIGURE 1:**
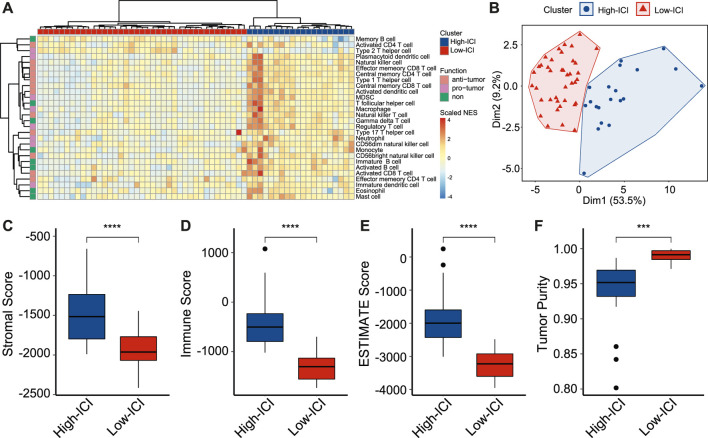
Landscape of immune cell infiltration in retinoblastoma. **(A)** Heatmap showing the normalized enrichment scores of each retinoblastoma sample on 28 immune cell types. **(B)** Scatter plot showing the distinct subgroups of retinoblastoma divided by PCA. **(C–F)** Boxplot showing the difference of stromal score **(C)**, immune score **(D)**, ESTIMATE score **(E)**, and tumor purity **(F)** between two retinoblastoma subtypes.

To study immune cell infiltration (ICI) of retinoblastoma, we performed the single-sample Gene Set Enrichment Analysis (ssGSEA) of 59 retinoblastoma patients from GSE58780 (Hänzelmann et al., 2013; Liu J. et al., 2021). Using gene sets which are related to 28 types of immune cells (Charoentong et al., 2017), the immune infiltration levels of all immune cell types were calculated (Figure 1A). Among these 28 types of immune cells, 12 types of immune cells, such as activated CD4 T cell and activated CD8 T cell, are considered to execute anti-tumor immunity; while 8 types of immune cells, such as regulatory T cell and type 2 T helper cell are considered having immune-suppressive functions (Jia et al., 2018). We used an unsupervised hierarchical clustering algorithm to assign the retinoblastoma samples into two clusters (high-ICI subgroup and low-ICI subgroup) based on immune infiltration levels (Figure 1A). The principal component analysis (PCA) of the retinoblastoma samples by immune infiltration levels confirmed the rationality of the result of hierarchical clustering (Figure 1B). The normalized enrichment score (NES) of each immune cell was then compared between the two subgroups, and the NES of 24 immune cells was significantly higher in the high-ICI subgroup (Supplementary Figure S1A). Subsequently, leveraging the ESTIMATE algorithm, we found that the stromal, immune, and ESTIMATE scores were relatively higher in the high-ICI subgroup (Figures 1C–E), whereas the tumor purity in the high-ICI subgroup was lower than that in the low-ICI subgroup (Figure 1F) (Yoshihara et al., 2013).

To prove that a higher ICI score represents a higher ICI level, we applied the ssGSEA to retinoblastoma scRNA-seq data from GSE174200 (Norrie et al., 2021), and the retinoblastoma samples were divided into two subgroups (Supplementary Figure S2A). Consistently, the high-ICI subgroup exhibited a higher immune cell percentage, indicating the robustness of the classification (Supplementary Figure S2B).

We next explored the differences in the clinical and pathological features between the two subgroups. The tumor diameter of the high-ICI subgroup was significantly smaller (mean diameter = 14.56 versus 15.8 mm, Supplementary Figure S1B). Patients with a high ICI score were significantly more likely to be hereditary forms (*RB1* mutation), whereas most of the patients with a low ICI score were non-hereditary (Supplementary Figure S1C). In the high-ICI subgroup, 60% of the patients were bilateral, higher than that in the low-ICI subgroup (Supplementary Figure S1D). Other characteristics (e.g., growth pattern, necrosis, optic nerve invasion, and choroid and sclera invasion) exhibited no statistical significance between the two subtypes (Supplementary Figures S1E–H).

Altogether, by estimating the immune infiltration levels in patients with retinoblastoma, we identified two different retinoblastoma subtypes with distinct immune features. We also found that the two subtypes exhibited significant differences in tumor size, *RB1* mutation, and laterality.

### The Two Subgroups Displayed Differences in the Expression of Photoreceptor Markers and Proliferation Genes

**FIGURE 2:**
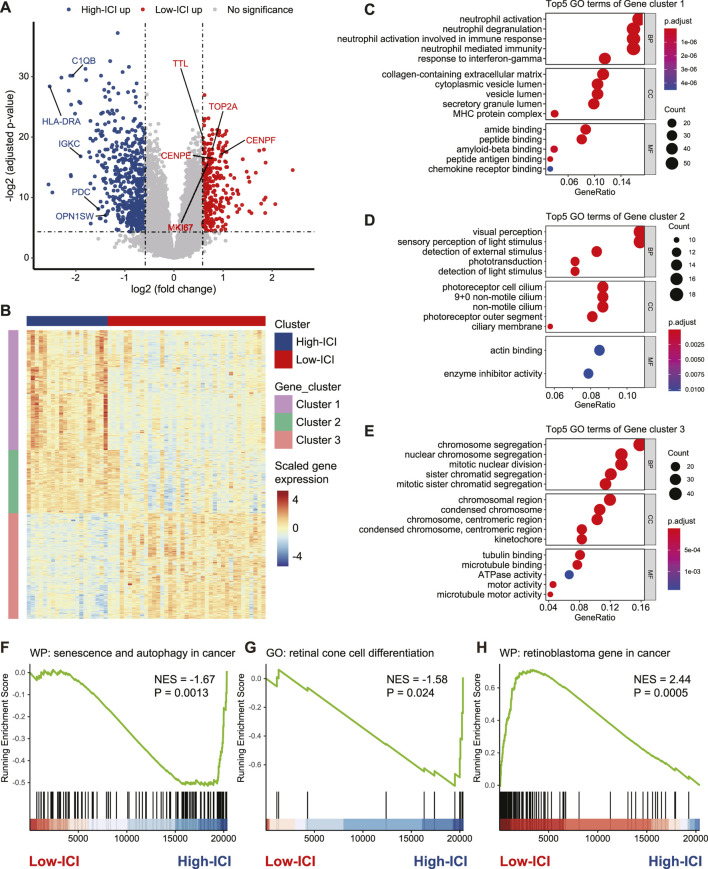
Identification and functional annotation of DEGs between two subtypes. **(A)** Volcano plot depicting the DEGs in two subtypes. Specific genes were indicated. **(B)** Heatmap of the gene expression value of DEGs on each sample. **(C–E)** Dot plots of the top-ranked GO terms of genes in gene cluster 1 **(C)**, gene cluster 2 **(D)**, and gene cluster 3 **(E)**. **(F–H)** Enrichment plot showing upregulation of the “senescence and autophagy in cancer” **(F)**, “retinal cone cell differentiation” **(G)**, and downregulation of the “retinoblastoma gene in cancer” (**H**) in the high-ICI subgroup.

To investigate the genes associated with immune cell infiltration, we performed a differential expression analysis to detect differentially expressed genes (DEGs) between the two subtypes by using the *limma* R package (Ritchie et al., 2015). Five-hundred fifty five and 320 genes were upregulated in the high-ICI subgroup and low-ICI subgroup, respectively (Figure 2A, Supplementary Table S1). Photoreceptor-specific genes, such as *OPN1SW* and *PDC*, were specifically expressed in the high-ICI subgroup, whereas proliferation markers (e.g., *MKI67*, *TOP2A*, *CENPE*, *CENPF*, and *TTL*) were highly expressed in the low-ICI subgroup (Figure 2A). By performing an unsupervised hierarchical clustering, these DEGs were classified into three main gene clusters, including two upregulated in the high-ICI subgroup and one upregulated in the low-ICI subgroup (Figure 2B). We next performed a Gene Ontology (GO) enrichment analysis of each gene cluster. The genes in cluster 1 were enriched for “visual perception,” “sensory perception of light stimulus,” and “phototransduction” (Figure 2C). The genes of cluster 2 were associated with immune/inflammation signature (e.g., “neutrophil activation,” “response to interferon-gamma,” and “MHC protein complex”) (Figure 2D). The genes of cluster 3 were related to “chromosome segregation,” “mitotic nuclear division,” and “tubulin binding” (Figure 2E). The enrichment analysis of these three gene clusters using other databases (KEGG, HALLMARK, PID, and REACTOME) exhibited enrichments in similar pathways (Supplementary Figures S3A–C). Altogether, our data showed that the low-ICI subgroup presented a high proliferation potential.

Next, we used a gene set enrichment analysis (GSEA) to obtain deeper insights into the function of immune infiltration in retinoblastoma. For example, the high-ICI subgroup was enriched for “senescence and autophagy in cancer” and “retinal cone cell differentiation” (Figures 2F,G). The low-ICI subgroup was enriched for “retinoblastoma gene in cancer” (Figure 2H). Taken together, our results suggest that retinoblastoma in the high-ICI subgroup maintains a cone-differentiation state, and the overexpression of proliferation markers which we found in the low-ICI subgroup may result in a higher propensity for metastasis.

### DNA Methylation Analysis Based on Different Immune Subtypes of Retinoblastoma

**FIGURE 3:**
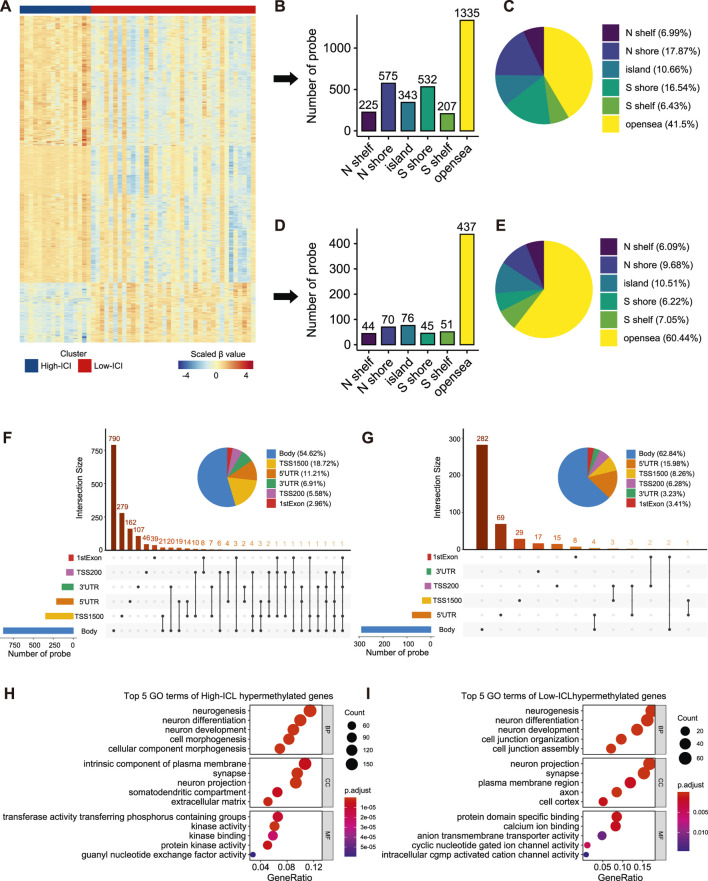
Differential methylation analysis of DNA methylation array data based on two subtypes. **(A)** Heatmap of the β value of DMPs on each sample. **(B)** Bar plot showing the number of high-ICI hypermethylated probes distributed to different regions. **(C)** Pie plot representing the composition of high-ICI hypermethylated probes. **(D)** Bar plot showing numbers of low-ICI hypermethylated probes distributed to different regions. **(E)** Pie plot representing the composition of low-ICI hypermethylated probe. **(F–G)** Upset graph showing the distribution of high-ICI **(F)** and low-ICI **(G)** hypermethylated probes on different genomic regions. Dot plots of the top-ranked GO terms of high-ICI hypermethylated genes **(H)** and low-ICI hypermethylated genes **(I)** are shown.

To interrogate the differences in the epigenome between the two immune subtypes of retinoblastoma, we analyzed DNA methylation with DNA methylation arrays from GSE58783. There were 16 and 37 cases corresponding to the high-ICI subgroup and low-ICI subgroup respectively. Leveraging the *ChAMP* R package, a total of 3,940 significantly differentially methylated probes (DMPs) were detected, including 3,217 and 723 hypermethylated probes in the high-ICI subgroup and low-ICI subgroup, respectively (Figure 3A, Supplementary Table S2) (Tian et al., 2017). Next, we explored the distributions of these DMPs. By considering the CpG island and the adjacent context, hypermethylated probes in the high-ICI subgroup were mostly located in the open sea (41.50%), followed by N-Shore (17.87%) and S-Shore (16.54%) (Figures 3B,C), while hypermethylated probes in the low-ICI subgroup were mostly located in the open sea (60.44%), followed by the CpG island (10.51%) and N-Shore (9.68%) (Figures 3D,E). After removing the probes in the intergenic region, the distributions of hypermethylated probes in the high-ICI subgroup in various functional genomic regions are shown in Figure 3F. The majority of the probes were discovered to be located in the gene body (54.62%), TSS1500 (200–1,500 bp upstream of the TSS, 18.72%) and 5′UTR (11.21%). The distributions of hypermethylated probes in the low-ICI subgroup were similar to those in the high-ICI subgroup, but some changes were observed. For example, the ratio of the probes across the gene body was higher (62.84%), but that across the 5′UTR area was lower (15.98%) (Figure 3G).

Next, we inquired about the potential biological processes related to DMPs. We mapped them to their corresponding genes and identified 1,519 hypermethylated genes in the high-ICI subgroup and 402 hypermethylated genes in the low-ICI subgroup (Supplementary Figure S4A). Surprisingly, by using an enrichment analysis, we observed that the hypermethylated genes in both subgroups were related to “neurogenesis,” “neuron differentiation,” and “neuron development” (Figures 3H,J). Moreover, some immune-related genes were included in the hypermethylated genes of the high-ICI subgroup, such as *CD83*, *HLA-DOA*, *IRF4*, *DOK3*, and *CXCR1*. Similarly, we also found that the hypermethylated genes of the high-ICI subgroup were significantly enriched for some biological processes related to immune regulation (e.g., T cell activation and B cell receptor signaling pathway), but no immune-related pathway was observed to be enriched by hypermethylated genes in the low-ICI subgroup (Supplementary Figure S4B). These results suggest that DNA hypermethylation is associated with the tumor immune microenvironment of retinoblastoma.

### Identification of Differentially Methylated and Expressed Genes and PPI Networks

**FIGURE 4:**
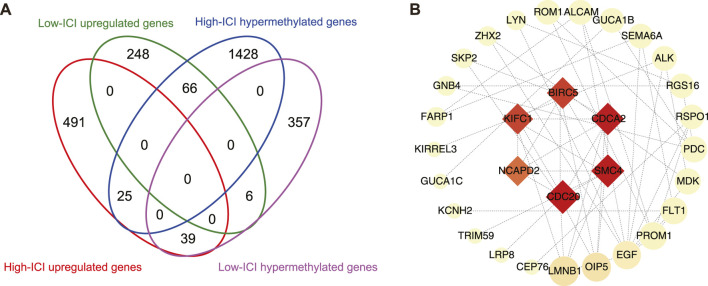
Protein–protein interaction network on differentially methylated and expressed genes. **(A)** Flower plot showing the overlap of DEGs and DMGs. **(B)** Protein–protein interaction (PPI) network of differentially methylated DEGs. Hub genes were presented at the middle of network. The color and area of nodes reflect the score which is calculated by *cytoHubba*.

Given that DNA methylation is a major epigenetic factor influencing gene expression, we further investigated which DEGs underwent methylation or demethylation (Moore et al., 2013). Subsequently, the overlapping of DEGs and differentially methylated genes (DMGs) was performed, then 66 and 39 hypermethylated downregulated genes were found in the high-ICI subgroup and low-ICI subgroup, respectively (Figure 4A). To further understand the underlying functions of differentially methylated and expressed genes, these genes were inputted into the STRING to build protein–protein interaction (PPI) networks (Szklarczyk et al., 2019). The PPI network consisted of 48 nodes and 66 edges. Using *cytoHubba*, we removed the nodes with a low connectivity score (less than 2), and the biggest module was retained (Figure 4B) (Chin et al., 2014). In this module, six nodes (*BIRC5*, *CDCA2*, *SMC4*, *CDC20*, *NCAPD2,* and *KIFC1*) had more than 20 connectivity scores, which were further screened and identified as hub genes.

## Discussion

Over the past several years, immune cell infiltration in tumors has been found to be of crucial importance in precision medicine, which can be attributed to the deep understanding of the tumor microenvironment (Yang, 2015; Tamborero et al., 2018). Immunotherapy has been successfully applied to various types of cancers (Havel et al., 2019). A recent study has proved the efficacy of immunotherapy in eliminating retinoblastoma cells whilst preserving the mouse vision (Wang et al., 2020). However, the treatment of advanced retinoblastoma remains challenging, thus, further therapeutic development is needed (Abramson et al., 2015). Therefore, elucidation and understanding of the immune landscape of tumors may not only provide insights into tumor immune dysregulation, but also lay the foundation for identifying novel immunotherapy targets.

In the present study, we characterized immune cell infiltration patterns in retinoblastoma using GEO databases. We used the ssGSEA method which is suitable for cross-platform evaluations of the landscape of 28 types of immune cells in retinoblastoma, and found two distinct subgroups. Patients with retinoblastoma in the high-ICI subgroup highly express late-stage cone markers (e.g. *GUCA1A* and *OPN1SW*) (Welby et al., 2017), whereas patients in the low-ICI subgroup highly express proliferation genes (e.g., *MKI67* and *TOP2A*) and retinoblastoma-related genes [e.g., *TTK* (Zeng et al., 2020) and *CDC25A* (Singh et al., 2015)], suggesting that immune cell infiltration is associated with retinoblastoma migration and metastatic progression. Consistent with the high expression of proliferation genes in patients with a low-ICI score, patients in the low-ICI subgroup exhibited a larger tumor diameter. Our study also showed that there was no significant difference in other clinical features (e.g. growth pattern, necrosis, optic nerve invasion, and choroid and sclera invasion) between the two subgroups. Further experiments are needed to confirm this result.

Many bioinformatics tools are being developed for the immune infiltration estimation of various cancer tissues based on their transcriptional data (Hänzelmann et al., 2013; Yoshihara et al., 2013; Newman et al., 2015). However, for validation, the application of a protein analysis on the single-cell level, including flow cytometry and immunostaining, on analyzed samples is required, which is essential to increase the reliability.

DNA methylation has proved its role as a significant epigenetic driving factor in cancer progression, development, and metastasis (Fleischer et al., 2014; Feinberg et al., 2016; Fleischer et al., 2017; Sina et al., 2019). Although evidence of DNA methylation regulating the immune microenvironment in breast cancer (Fleischer et al., 2017), glioma (Briand et al., 2019), and gingivo-buccal oral cancer (Das et al., 2019) has been presented, the role of DNA methylation in the retinoblastoma immune environment has not been completely explored. We systematically analyzed DNA methylation based on the two subgroups. Further analysis of DNA methylation differences between the two subgroups of retinoblastoma patients showed that a global DNA hypermethylation pattern was presented in the high-ICI subgroup (3,217 hypermethylated probes versus 723 hypomethylated probes). The enrichment analysis of these differentially methylated genes showed that these genes were remarkably related to the immune regulation in the tumor microenvironment including T cell activation, B cell receptor signaling pathway, and cytokine signaling in the immune system. These results implied that alterations in DNA methylation may play a crucial role in retinoblastoma immune cell infiltration.

The PPI network of differentially expressed and methylated genes provided a comprehensive observation of their functional connections, and screened hub genes. We identified a total of six hub genes: *BIRC5*, *CDCA2*, *SMC4*, *CDC20*, *NCAPD2,* and *KIFC1*. Among the hub genes, *BIRC5* (also named survivin) is a well-known cancer therapeutic target (Li et al., 2019). *BIRC5* immunotherapy-related clinical trials have been applied in patients with colorectal cancer (Tsuruma et al., 2004), malignant glioma (Fenstermaker et al., 2016), and melanoma (Becker et al., 2012). The role of *BIRC5* in retinoblastoma has also been investigated before. Exposure to carboplatin, topotecan, or radiation resulted in the elevated expression of *BIRC5* in the retinoblastoma cell line (Ferrario et al., 2016). A previous study showed that Dnmt1, Dnmt3a, and Dnmt3b can regulate the methylation status of *BIRC5* in glioblastoma multiforme (Hervouet et al., 2010). Our result extended and enriched the knowledge about the relationship between *BIRC5* and DNA methylation in tumors.

This study is not devoid of limitations. First, all our results were theoretical and validation based on patients or animal samples is lacking. Second, we had not found another dataset that includes DNA methylation data, so a validation cohort to confirm our conclusions is needed. Third, gene expression is a complex process involving numerous steps and many other regulatory elements, such as DNA methylation, nucleosome positioning and composition, 3D structural interactions, and histone modification can alter gene expression (Carter and Zhao, 2021). However, because of the lack of other multi-omics data, here we only elaborated on the relationship between gene expression and DNA methylation. Fourth, we only focused on hypermethylated downregulated genes and hypomethylated upregulated genes, which is a generally accepted regulative paradigm between DNA methylation and gene expression (Esteller, 2002; Ehrlich, 2009; Witte et al., 2014). However, recent efforts identified subtle changes in the relationship between DNA methylation and gene expression, beyond the classical dogma (Wan et al., 2015; Liu A. et al., 2021). Therefore, further analysis is required to evaluate contra-regulated genes. Fifth, we did not apply single-cell-level protein profiling, which is able to provide solid evidence to measure the infiltration level, on retinoblastoma to investigate the infiltration pattern.

In summary, by comprehensively assessing the immune cell infiltration in retinoblastoma, we highlight the differences between the two subgroups in gene expression and DNA methylation levels. The retinoblastoma immune landscape analysis may help clinicians develop novel immunotherapeutic targets.

## Data Availability

Publicly available datasets were analyzed in this study. These data can be found at: GSE58780 GSE58783 GSE174200.

## Supplementary Material

Supplementary Figure S1 Immune scores and clinical characteristics between two retinoblastoma subtypes. **(A)** Box plot showing the comparison of two subtypes in terms of their NES of different immune cell types. **(B‐H)** Bar plot representing composition of **(B)** tumor diameter, **(C)** RB1 germ line, **(D)** laterality, **(E)** growth pattern, **(F)** necrosis, **(G)** optic nerve invasion, and **(H)** choroid and sclera invasion in the high-ICI and the low-ICI subgroup.

Supplementary Figure S2 Immune cell infiltration landscape of scRNA-seq data from retinoblastoma patients. **(A)** Heatmap showing the normalized enrichment scores of scRNA-seq data from retinoblastoma patients on 28 immune cell types. **(B)** Box plot showing the difference in the immune cell percentage between two retinoblastoma subtypes.

Supplementary Figure S3 Enrichment pathways of DEGs. **(A–C)** Dot plot showing the enriched pathways of gene clusters, **(A)** gene cluster 1, **(B)** gene cluster 2, and **(C)** gene cluster 3.

Supplementary Figure S4 Functional annotation of DMGs. **(A)** Volcano plot depicting the DMGs in two subtypes. Specific genes were indicated. **(B)** Dot plot showing the enriched pathways of high-ICI hypermethylated genes, and immune-related pathways were selected.
