# Supplementary figures and images for: Comprehensive Analysis of the Immune Cell Infiltration Landscape and Immune-Related Methylation in Retinoblastoma
Source: Front Genet. 2022 May 18;13:864473. doi: 10.3389/fgene.2022.864473 (PMC9157546; doi:10.3389/fgene.2022.864473)

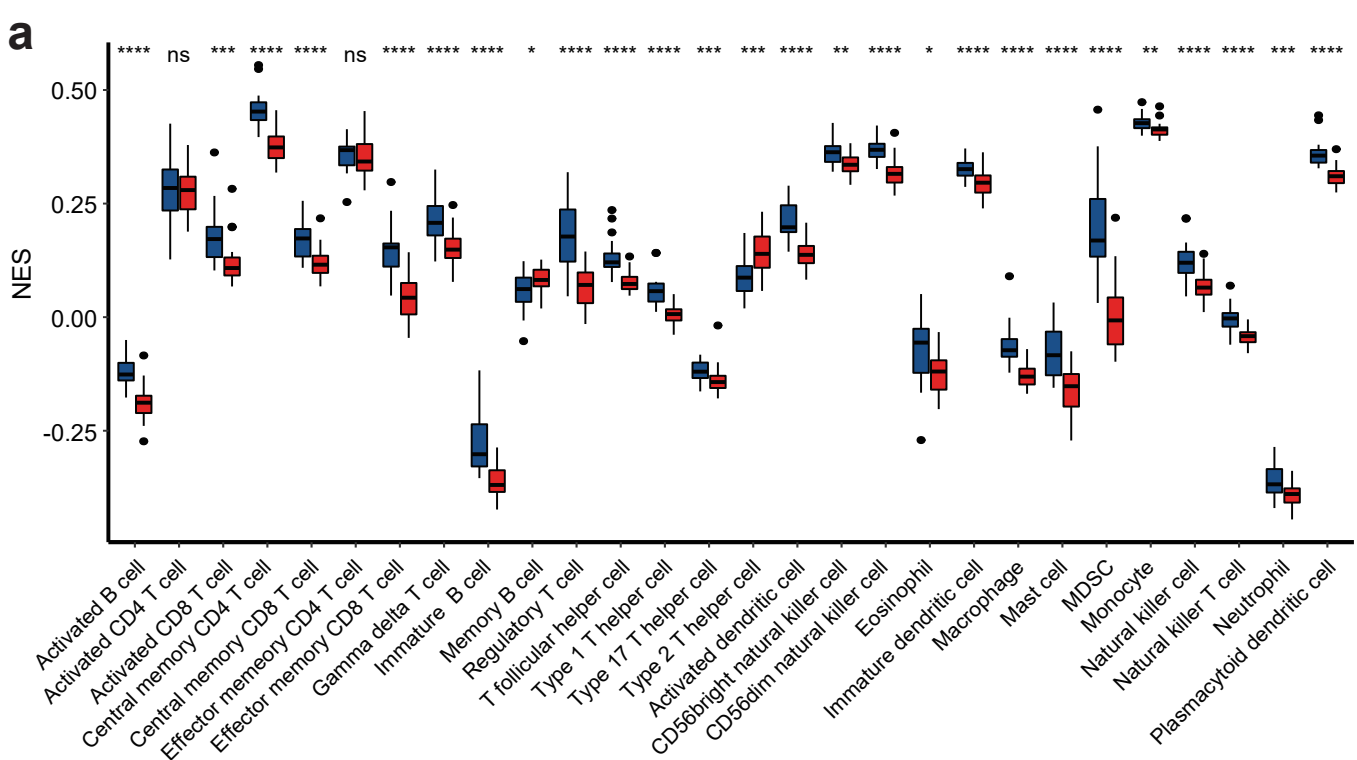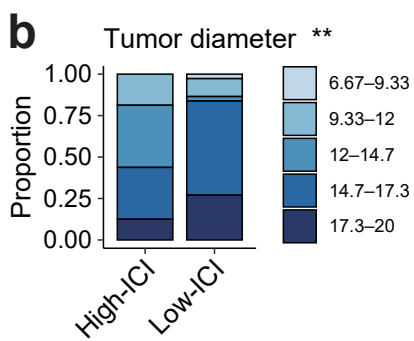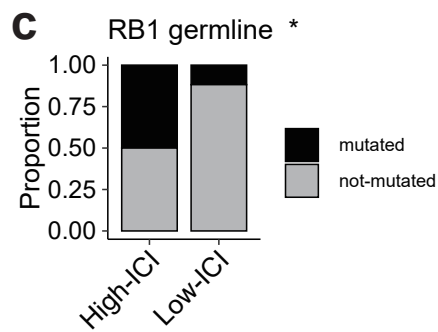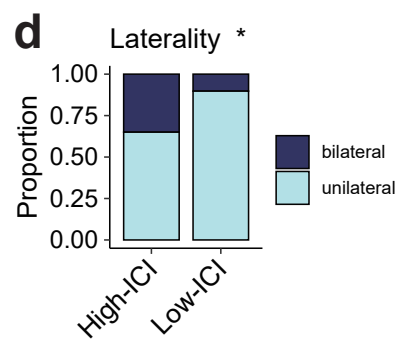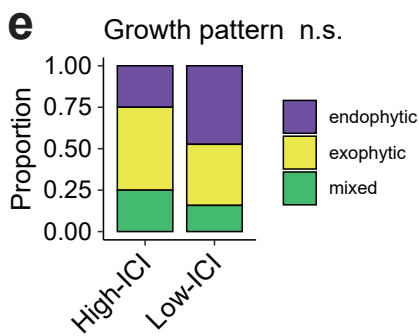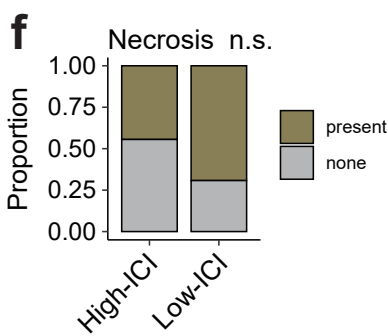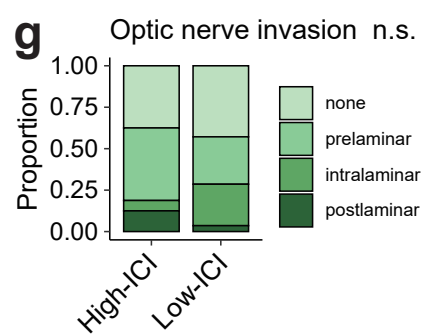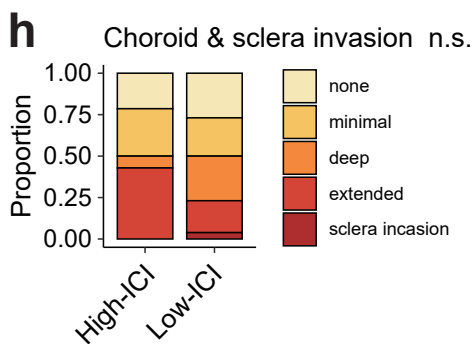

**a**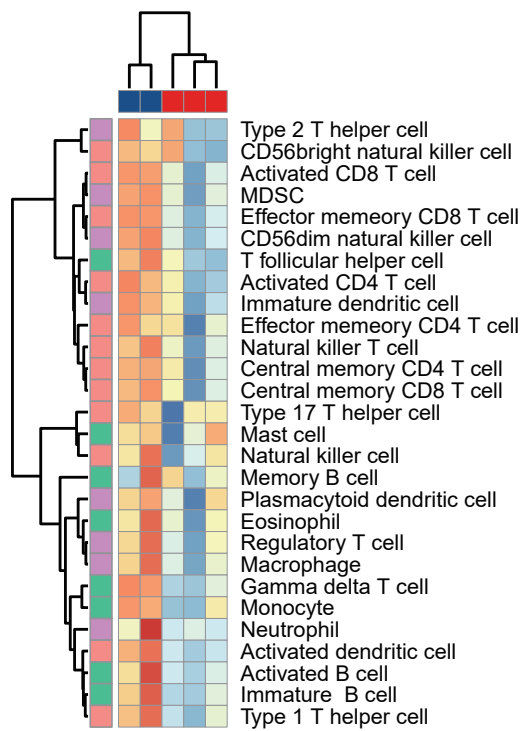**b**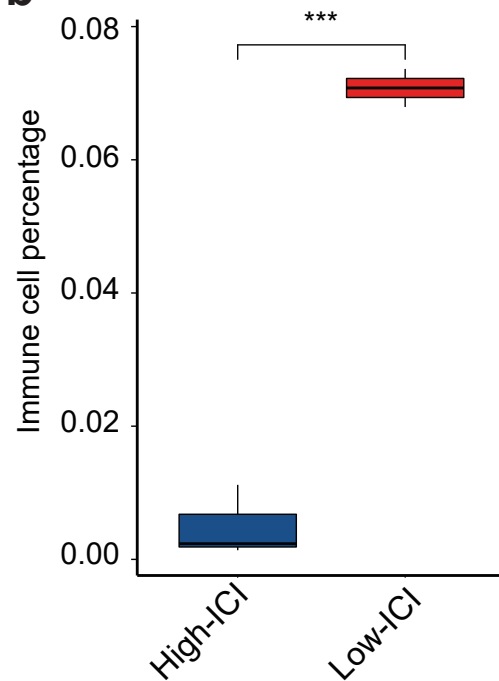

**a**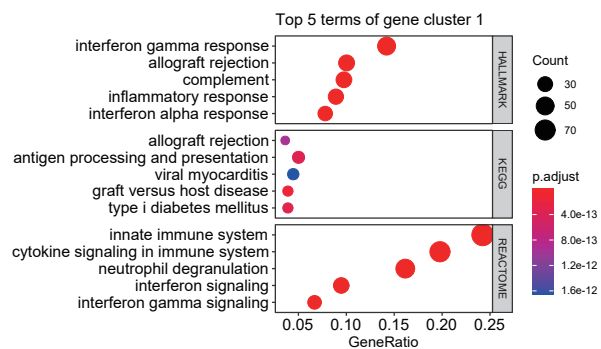**b**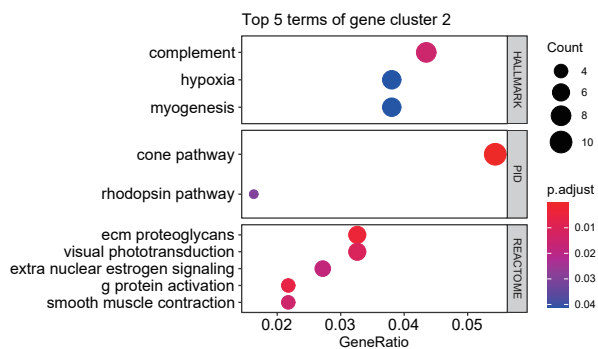**c**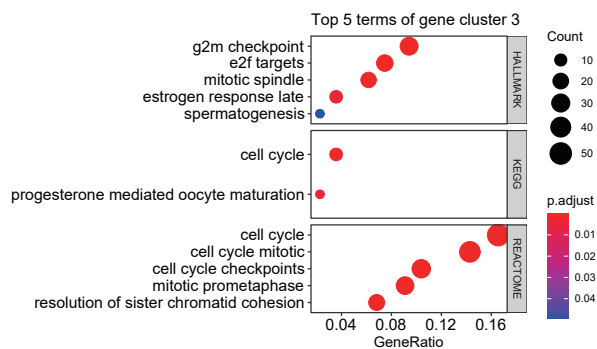

**a**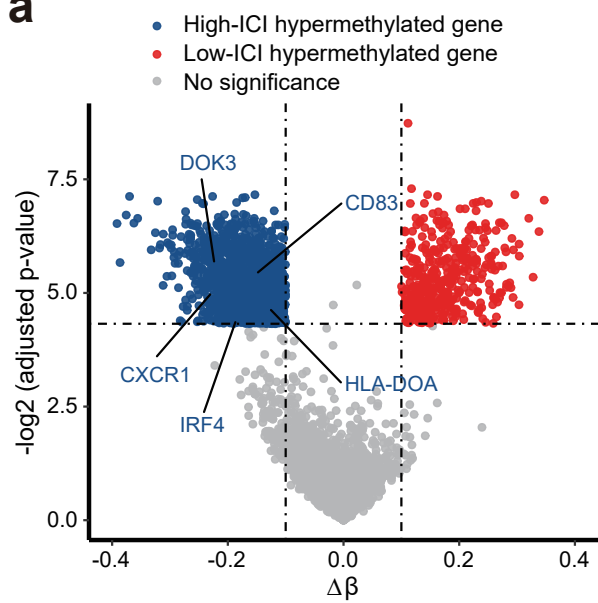**b**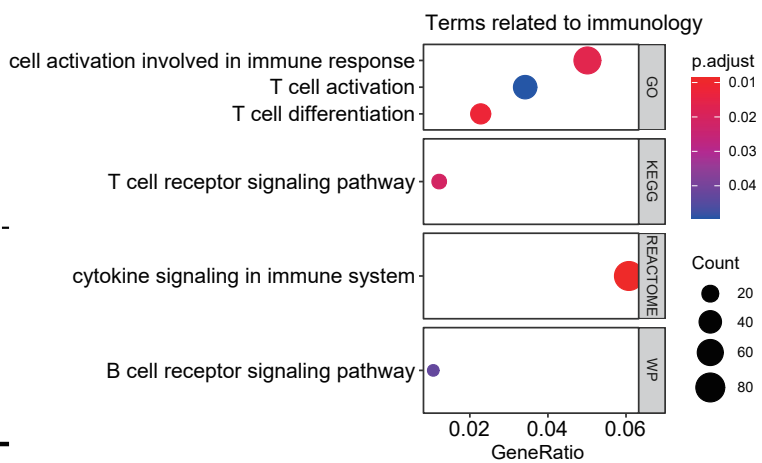

Supplement: Supplementary file 1 [file DataSheet1.pdf]
